# A case report of Arnold Chiari type 1 malformation in acromesomelic dwarf infant

**DOI:** 10.11604/pamj.2021.38.58.27295

**Published:** 2021-01-18

**Authors:** Miteshkumar Rajaram Maurya, Renju Ravi, Sona Ajit Pungavkar

**Affiliations:** 1 Department of Clinical Pharmacology, Seth Gordhandas Sunderdas Medical College and King Edward Memorial Hospital, Mumbai, Maharashtra, India,; 2 Siddhi Diagnostic and Research Centre, Nallasopara West, Maharashtra, India

**Keywords:** Arnold Chiari 1 malformation, acromesomelic dwarfism, hydrocephalus, neural tube defects, case report

## Abstract

Arnold Chiari malformation is one of the commonest cause of congenital hydrocephalus. Cause of fetal development of cerebellar tonsils remains unknown and may be diagnosed at later in life. The association of Arnold Chiari malformation with acromesomelic dwarfism is not known. We report male infant diagnosed with acromesomelic dwarfism at end of gestation period on basis of antenatal ultrasonography findings. An ultrasound scan of infant head at fifth month of birth was performed in view of increasing head circumference that revealed aqueductal stenosis with dilated posterior horn of lateral ventricles in brain.

## Introduction

Arnold Chiari malformations were first described in pediatric autopsy specimen in 1891 by Hans Chiari, an Austrian Pathologist (1851-1916) [1]. In legacy with name of his professor Dr. Arnold and his name Hans Chiari, the hind brain disorder is named as Arnold Chiari malformation [2]. Exact etiology remains unknown. Arnold Chiari type I malformation remains mostly asymptomatic until adulthood. Diagnosis is made by measuring the length of protrusion of cerebellar tonsils below the inner margins of foramen magnum to the inferior most part of tonsils (measurement taken from ophisthion to basion). If tonsils are above the foramen magnum considered as normal, if tonsillar length is <5mm is also normal but the term benign tonsillar ectopia can be used and if tonsillar length is >5 mm then it is termed as Arnold Chiari type I malformation [3]. Incidence of Arnold Chiari malformation is gradually increasing because of increased detection with various imaging techniques available. Hydrocephalus is one of the common presentation of Arnold Chiari 1 malformation.

## Patient and observation

A 5-month-old baby weighing 2.6 kg at birth and born of non-consanguineous marriage presented to the pediatrician with increase in the head circumference. The child was feeding well and there were no other complaints. At the ninth month of pregnancy, the child has been diagnosed with acromesomelic dwarfism during a routine antenatal scan. The mother gave a history of the first child having severe hydrocephalus with undeveloped crania (on intrauterine sonogram) at 19 weeks of gestation. The pregnancy was terminated. The mother was subsequently put on the multi-vitamin and folic acid tablet supplementation before the second conception. The second conception occurred 2.5 years later.

**Figure 1:**
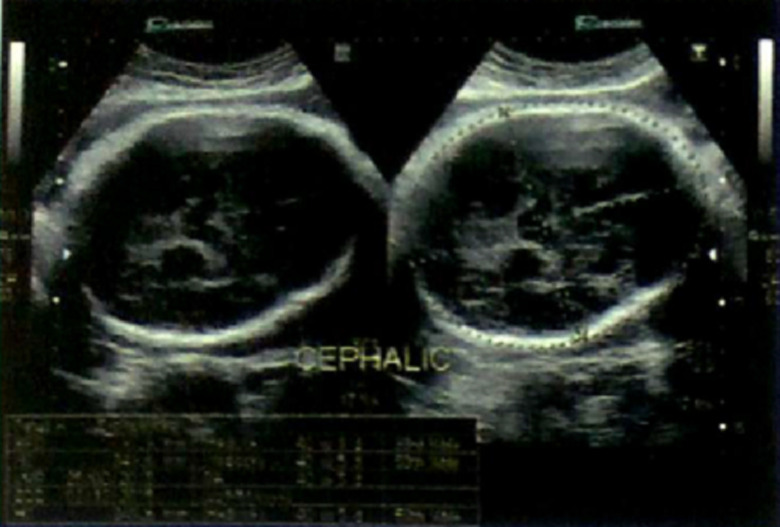
antenatal ultrasound scan of brain

**Figure 2:**
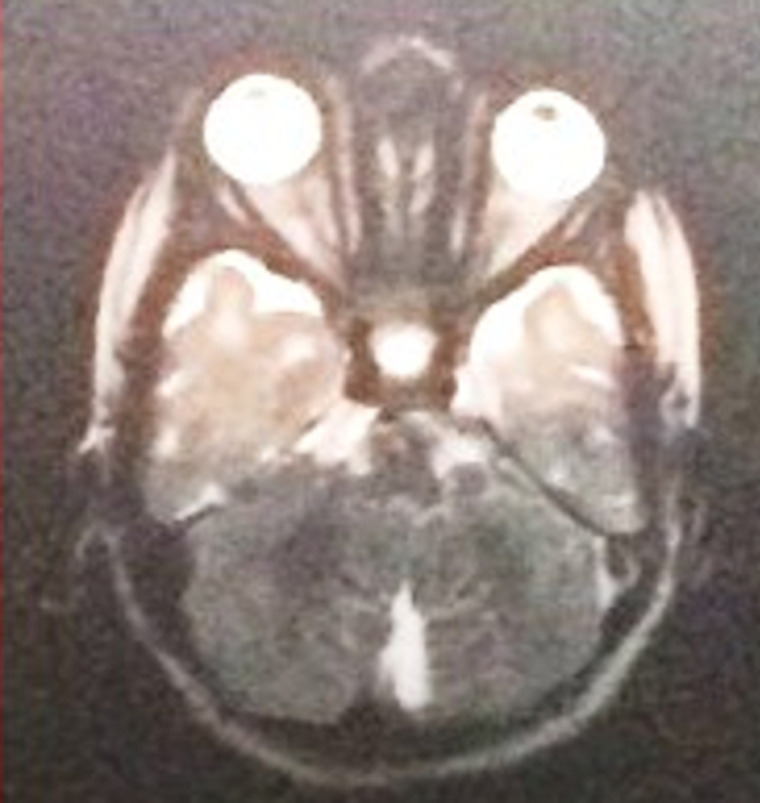
magnetic resonance imaging (MRI) of the brain

**Figure 3:**
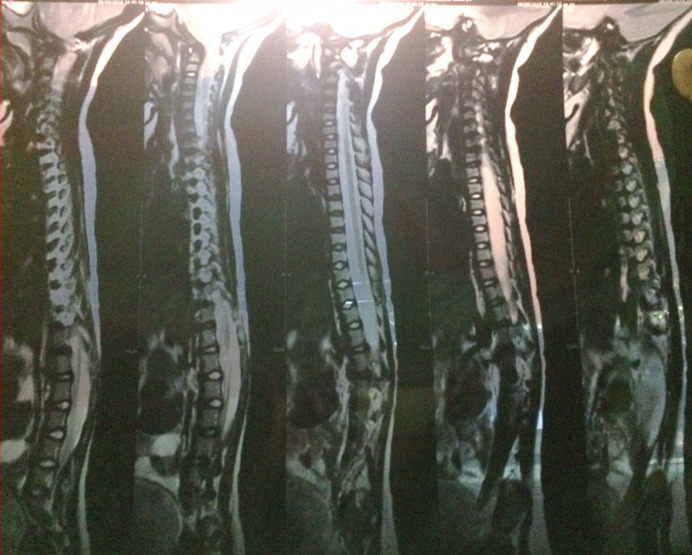
magnetic resonance imaging (MRI) scan screening of whole spine

An ultrasound scan of head done at fifth month of birth (Figure 1) showed ventriculomegaly with dilatation of posterior horn (transverse diameter of 2.4cm). The third and lateral ventricles appeared dilated (anterior horn of lateral ventricle on left side measured 23mm in width and the posterior horn measured-24mm). The fourth ventricles were normal. No intra-cranial or intra-ventricular/germinal matrix hematoma was detected. The brain parenchyma was normal as were the sulci and gyri and frontal, temporal and parietal lobes. No subdural or extradural collection was seen. A provisional diagnosis was made of infantile hydrocephalus or aqueductal stenosis and a MRI brain with screening of whole spine was advised by pediatrician. MRI scan brain with screening of whole spine was done at the fifth month of birth that revealed herniation of cerebellum tonsil (4mm) through the foramen magnum into the upper cervical canal consistent with Arnold Chiari type 1 malformation with no evidence of syrinx formation or cord edema (Figure 2 and Figure 3). There was compression of the cervico-medullary junction with mild to moderate obstructive hydrocephalus, without periventricular ooze. There was atrophy of both lentiform nuclei with paucity of white matter in bilateral occipital regions with thinning of the corpus callosum. There was rounded 3 mm distended subarachnoid spaces around the optic nerves. Cerebral sulci well seen. Aqueduct canal was well formed. Evidence of occluded subarachnoid spaces at foramen magnum was present. There was no evidence of neural tube defects.

Next generation sequencing (NGS) (Table 1) revealed presence of heterozygous LAMB1 (encodes for laminin subunit beta-1 protein) and MPDZ gene (encodes for Multiple PDZ domain protein) mutation from paternal side whereas that of GAA gene (encodes for acid alpha-glucosidase also known as acid maltase synthesized in lysosomes) mutation from maternal side (gene associated with congenital hydrocephalus). LAMB1, MPDZ were analyzed because the mutations in either are known to be associated with congenital hydrocephalus with autosomal recessive genetic inheritance. The mutation in fibroblast growth factor receptor 3 (FGFR3) gene responsible for rhizomelic short stature was present in infant producing clinical picture of achondroplasia, which is autosomal dominant genetic condition. Definitive prenatal diagnosis can be offered at present only for achondroplasia gene (FGFR3) mutation, the empiric recurrence risk being around 5% (to account for possibility of germinal mosaicism). A neurosurgery consultation was done and ventriculo-peritoneal shunt or third ventriculostomy or suboccipital craniectomy with first and second vertebra laminectomy was advised. However, the guardian of patient were reluctant to go for this procedure due to unsurity of benefits and repeat surgery need as explained to them.

**Table 1:** genetic work up for infant diagnosed with rhizomelic short stature and hydrocephalus; infant was diagnosed with heterozygous mutation in following genes in next generation sequencing (NGS) for FGFR3, MPDZ, LAMB1, GAA gene

Sr no	Gene	Variant	Genotype	Coverpage	dbSNPid	Interpretation	Phenotype
1	FGFR 3	Chr4:1806119:G>A NM_000142:exon9:e.G1138A:p.G380R	Het	93x	rs28931614	*P	Achondroplasia: ACH
2	MPDZ	Chr9:13223709:C>T NM_001261406:exon5:e.G394A:p.G132S	Het	111x	rs201101621	#VUS	Hydrocephalus, nonsyndromic, autosomal recessive 2
3	LAMB1	Chr7:107575915:A>T:NM_002291:exon27:e.T4133A:pL1378H	Het	131x	rs769712243	#VUS	Lissencephaly 5
4	GAA	Chr17:78078386:A>G NM_000152:exon2:e.A1G:p.M1V	Het	33x	rs786204467	*P	Pompe Disease

* P: pathogenic, #VUS: variant of uncertain significance; Het: heterozygous

## Discussion

This paper describes a case of an infant presenting with progressive increase in head circumference at fifth month of gestation and diagnosed with Arnold Chiari malformation type 1. There are only isolated reports of Arnold Chiari type 1 malformation or acromesomelic dwarfism in the Indian population. Very few cases present with Arnold Chiari type 1 malformation in association with acromesomelic dwarfism and progression of hydrocephalus [4-7]. Danda S *et al*. study reported about a family siblings affected with acromesomelic dwarfism [4]. Haldar *et al*. described about the anesthesia concerns involved in patients with Acromesomelic dysplasia with associated hydrocephalus, Arnold Chiari malformation and syringomyelia [5]. Arnold Chiari malformation is almost always associated with neural tube defects [6].

The exact etiology of Arnold Chiari malformation and Acromesomelic short stature remains unknown. A large metanalysis strongly suggests that the MTHFD 1 G1958A gene polymorphism is strongly linked with neural tube defect [7]. Urbizu *et al*. identified four genetic variants (located in the genes ALDH1A2, CDX1 and FLT1) to be associated with adult classic Chiari malformation type 1 [8]. Markunas *et al*. identified different levels of expression in genes related with dorso-ventral axis formation (ETS1, ETS2, NOTCH4), ribosome, spliceosome and proteasome in pediatric classic Chiari malformation type 1 patients [9]. There is limited information on MTHFR (methylene tetrahydrofolate reductase) gene mutation prevalent among the Indian population that may interfere with benefit of folic acid supplementation. Methylene Tetrahydrofolate Dehydrogenase gene (MTHFD) is also one of the key genes involved in folate metabolism pathway.

The fundamental challenge lies in early radiological diagnosis and detection of cases of dwarfism (Acromesomelic type) as well as Arnold Chiari 1 malformation. Also there exist lack of awareness about the branch of fetal medicine and perinatalogists who have expertise diagnosing the fetal malformations doing genetic workup for the same. Further evaluation need to be done for the pathophysiology of this disorder (viral origin/ drug induced/deficiency disorders/environmental factors/genetics) facilitating early prevention and appropriate intervention.

## Conclusion

Our study presents lately diagnosed case of Arnold Chiari I malformation in an infant after birth. However, we do not know if there is any association between Acromesomelic dwarfism and Arnold Chiari malformation. There is need to create awareness among the people about the importance of early antenatal anomaly scan and genetic screening with emerging branch of fetal medicine experts to diagnose early the occurrence of such malformations.
